# Factors potentially contributing to the decline of the mpox outbreak in the Netherlands, 2022 and 2023

**DOI:** 10.2807/1560-7917.ES.2024.29.21.2300608

**Published:** 2024-05-23

**Authors:** Manon R Haverkate, Inge JM Willemstein, Catharina E van Ewijk, Philippe CG Adam, Susan J Lanooij, Petra Jonker-Jorna, Colette van Bokhoven, Gini GC van Rijckevorsel, Elske Hoornenborg, Silke David, Liesbeth Mollema, Margreet J te Wierik, Jente Lange, Eelco Franz, Hester E de Melker, Eline LM Op de Coul, Susan JM Hahné

**Affiliations:** 1 National Institute for Public Health and the Environment, Bilthoven, the Netherlands; 2 European Programme for Intervention Epidemiology Training (EPIET), European Centre for Disease Prevention and Control (ECDC), Stockholm, Sweden; 3 Institute for Prevention and Social Research, Utrecht, the Netherlands; 4 University of New South Wales Sydney, Sydney, Australia; 5 Public Health Service Hollands Noorden, Alkmaar, the Netherlands; 6 Public Health Service Gelderland-Zuid, Nijmegen, the Netherlands; 7 Public Health Service Amsterdam, Amsterdam, the Netherlands

**Keywords:** mpox, monkeypox, disease outbreaks, primary preventive vaccination, behaviour, risk groups, sexually transmitted infections

## Abstract

**Background:**

In 2022 and 2023, a global outbreak of mpox affected mostly gay, bisexual and other men having sex with men (GBMSM). Outbreak control in the Netherlands included isolation, quarantine, post-exposure prophylaxis vaccination and primary preventive vaccination (PPV).

**Aim:**

We describe the course of the outbreak, the vaccination programme, vaccine effectiveness (VE) of full vaccination against symptomatic disease, and trends in behaviour to generate hypotheses about factors that influenced the outbreak’s decline.

**Methods:**

In this observational study, we collected data from public health services on notified cases, number of PPV invitations and PPV doses administered. We calculated PPV uptake and coverage. Trends in behavioural data of GBMSM visiting sexual health centres were analysed for all consultations in 2022. We estimated VE using the screening method.

**Results:**

Until 31 December 2023, 1,294 mpox cases were reported. The outbreak peaked in early July 2022 and then declined sharply. PPV started on 25 July 2022; in total 29,851 doses were administered, 45.8% received at least one dose, 35.4% were fully vaccinated. The estimated VE was 68.2% (95% CI 4.3–89.5%). We did not observe an evident decrease in high-risk behaviour.

**Discussion:**

It is unlikely that PPV was a driver of the outbreak’s decline, as incidence started to decline well before the start of the PPV programme. The possible impact of behavioural change could not be demonstrated with the available indicators, however, the data had limitations, hampering interpretation. We hypothesise that infection-induced immunity in high-risk groups was an important factor explaining the decline.

**Key public health message**

**What did you want to address in this study and why?**

In 2022 and 2023, a global outbreak of mpox among gay, bisexual and other men having sex with men (GBMSM) occurred. It peaked in summer 2022 and then declined rapidly. We wanted to know what factors may have contributed to the decline of the outbreak in the Netherlands. Furthermore, we estimated the vaccine effectiveness of preventive vaccination against symptomatic mpox.

**What have we learnt from this study?**

It is not likely that preventive vaccination was a driver of the outbreak’s decline, as the mpox incidence started to decline well before the start of the vaccination programme. Rather, the reason appears to have been that GBMSM subgroups at highest risk acquired immunity through infection and post-exposure prophylaxis and/or that they reduced their risk behaviour. Vaccine effectiveness of preventive vaccination was estimated at 68%.

**What are the implications of your findings for public health?**

To be effective in preventing new cases, future vaccination programmes need to be implemented fast after the first cases are observed. Surveillance of mpox, monitoring of risk behaviour and evaluation of vaccination programmes, including vaccine effectiveness, are important for prevention and control of future mpox outbreaks.

## Introduction

Since the first reported human case of mpox in 1970 in the Democratic Republic of the Congo, endemic transmission of monkeypox virus (MPXV) was for decades only reported from African countries. Sporadic travel-associated cases were described outside the African continent [1,2] and in 2003, an outbreak in the United States (US) was caused by imported mammals [3-5]. However, a global outbreak of mpox occurred in 2022, affecting mostly gay, bisexual and other men who have sex with men (GBMSM) [1,6,7]. Until 31 December 2023, there were more than 93,000 confirmed cases worldwide in 117 countries [8]. In the Netherlands, the first person with mpox was diagnosed on 20 May 2022, and the outbreak peaked in the beginning of July 2022. The first 1,000 cases in the outbreak of mpox in the Netherlands until 8 August 2022 have been described by van Ewijk et al. [9]. Since then, the incidence has continued to decrease. Between 8 August 2022 and 31 December 2023, an additional 294 people with laboratory-confirmed mpox were reported to the Dutch National Institute for Public Health and the Environment (RIVM), resulting in a total of 1,294 confirmed mpox cases. Only 34 of those 1,294 infections were reported in 2023.

Outbreak control measures in the Netherlands included isolation of persons with mpox and quarantine (later replaced by lifestyle recommendations). High- and medium-risk contacts of all persons diagnosed with mpox were offered post-exposure prophylaxis (PEP) vaccination with Modified Vaccinia Ankara-Bavarian Nordic (MVA-BN; also known as Imvanex or Jynneos), a third-generation smallpox vaccine [10]. A primary preventive vaccination (PPV) programme with MVA-BN started on 25 July 2022, targeting those at highest risk of infection [11]. Vaccination against smallpox had ceased in 1976 in the Netherlands [12] and worldwide in 1980 [13]. It is estimated that at the start of the 2022 mpox outbreak, 57% [9] to 68% [13] of the Dutch population did not have protective antibodies against orthopoxviruses. Starting dates for the PPV programme varied between different regions, but all regions started before 11 August 2022. Furthermore, general public communication started in May 2022, and a targeted communication campaign was initiated on 21 July 2022, alerting GBMSM and transgender persons of behaviours that would put them at higher risk of mpox and advising them about preventive measures to mitigate the risk [14]. 

Here, we describe the mpox outbreak in the Netherlands, the design and implementation of the vaccination programme, trends in behavioural data and MVA-BN vaccine effectiveness to generate hypotheses about the factors that may have contributed to the decline of the outbreak in the Netherlands.

## Methods

### Data collection

#### Epidemiology

All persons diagnosed with mpox were reported to the RIVM by the public health services (PHS) in an online reporting system as part of the mandatory notification [15]. In the Supplement, we append additional details on the different notification categories and corresponding regulations. For all those diagnosed with mpox, we collected information on, among other variables, demographic data, sexual orientation, potential source(s) of infection and results of contact tracing. A detailed description of the variables collected can be found in van Ewijk et al. [9]. Data on notifications were included until 31 December 2023.

#### Vaccination

The following groups were eligible for PPV: 1a. GBMSM and transgender persons using HIV-pre-exposure prophylaxis (PrEP) via sexual health centres (SHC); 1b. GBMSM and transgender persons on the waiting list for the HIV-PrEP-pilot via SHC; 1c. GBMSM and transgender persons using HIV-PrEP via their general practitioner (GP); 2. GBMSM and transgender persons living with HIV at high risk of mpox infection; 3. Other GBMSM and transgender persons at high risk of mpox infection [11]. All PHS identified the persons eligible and invited them individually for PPV. All vaccinations were given subcutaneously. The estimated numbers of PPV invitations sent out per indication group and all administered PPV doses were registered by all PHS in a dedicated online reporting system. For vaccinated persons who did not give consent to share their vaccination information with the RIVM, we only collected the following anonymised data: year of birth in three categories (< 1985, 1985–1994, > 1994), childhood smallpox vaccination (yes/no), PPV vaccination number (first or second), week of vaccination and PHS location. For persons who gave consent to share pseudonymised data, this was supplemented with: unique identifier, year of birth, gender (man/woman/other (non-binary, genderfluid, agender etc.)), indication group, immunocompromised (yes/no), received PEP vaccination (yes/no), date of PEP vaccination and date of PPV. Administered PEP vaccinations were not registered on a national level. Data on vaccinated persons were included until 30 April 2023. Details on PEP vaccination, the PPV programme and selection of persons eligible for PPV can be found in the Supplement.

#### Behavioural change

In the Netherlands, data regarding all visits to SHC are available in a national surveillance database (SOAP) of the RIVM. This database contains pseudonymised data from all consultations, including behavioural data. Consultations can be divided into regular SHC consultations and HIV-PrEP pilot consultations. Persons participating in the HIV-PrEP pilot are regularly tested for STIs at SHC (every 3 months), irrespective of their sexual behaviour. Monitoring behaviour in this group will therefore give a more accurate representation of possible changes in behaviour, compared with persons who consult an SHC because of symptoms or possible risk incurred. Therefore, we only included HIV-PrEP pilot consultations of GBMSM and transgender persons (including non-binary persons). Behavioural data were collected from all SHC consultations in 2022. As a person probably had multiple consultations in 2022, analyses of behavioural variables were done on consultation level. The variables we extracted from the SOAP database and used as proxies for behaviour with high risk of mpox infection were: number of sex partners, group sex (yes/no; defined as multiple sex partners at once or over a short period), chemsex (yes/no; defined as using drugs (cocaine, XTC/MDMA/speed, heroin, crystal meth, mephedrone, 3-MMC, 4-MEC, 4-FA, GHB/GBL and/or ketamine) before or during sex), condomless anal sex (always condomless vs sometimes/never; receptive and/or insertive) and sex work (yes/no). All behavioural variables are self-reported behaviour in the past 6 months. Furthermore, we collected total number of tests and percentage of test positivity for chlamydia, gonorrhoea and syphilis in 2022.

### Data analyses

#### Epidemiology

For persons diagnosed with mpox, we assumed people born ≥ 1976 in the Netherlands or ≥ 1980 in another country had not received a childhood smallpox vaccination.

#### Vaccination

We defined mpox vaccine uptake as the proportion of invited people receiving at least one dose, and mpox vaccination coverage as the proportion of invited people being fully vaccinated. A person was considered fully vaccinated if they had received two MVA-BN vaccinations; or if they received one MVA-BN vaccination and a childhood smallpox vaccination and were not reported to be immunocompromised. For the mpox vaccinees we had no information on country of birth. We assumed that people born < 1976 with an unknown childhood smallpox vaccination status had received this vaccination as a child, while childhood smallpox vaccination status was set to ‘No’ for everybody born ≥ 1980. For those born from 1976 to 1979, we used smallpox vaccination status as reported (including ‘unknown’). If it was unknown if a person was immunocompromised, we assumed they were not. For the analyses of the uptake and vaccination coverage, we excluded vaccinations administered to people who were not in the predefined indication groups. We calculated uptake and vaccination coverage for all invitees, and by indication group.

#### Vaccine effectiveness

We estimated vaccine effectiveness (VE) against laboratory-confirmed symptomatic mpox disease for those who were fully vaccinated using the screening method as described by Farrington [16]. This method compares the vaccination coverage in the cases with the coverage in the population they arose from, with the VE calculated as 1 – odds of vaccination in cases/odds of vaccination in the population. Cases were included in the analyses if they were male at birth, indicated that they had sex with men, and if their vaccination status was known (or, if they were born ≥ 1976 in the Netherlands or ≥ 1980 in another country, we assumed they had not received a childhood smallpox vaccination). For these analyses, we considered persons as fully vaccinated using the definition described above, plus their last vaccination should be at least 14 days before symptom onset to allow time for vaccine-induced protection [17]. Also, at least one of the vaccinations received should be a PPV, to make the cases representative for the population where the vaccination coverage was calculated from. Cases that had received their last vaccination less than 14 days before onset of symptoms were regarded incompletely vaccinated and were included in the total number of cases, but not in the completely vaccinated cases. We calculated the proportion of fully vaccinated among the cases per week. The vaccination coverage in the population (i.e. the proportion of fully vaccinated persons among those invited for PPV) was calculated per week as well. Also here, we considered a 14-day delay to allow time for vaccine-induced protection. Increasing vaccination coverage was accounted for by fitting a logistic regression model with the logit of the proportion of the population vaccinated as offset [16]. To reduce bias introduced by the small sample size, we applied Firth correction to the logistic regression model. As the number of mpox infections dropped dramatically by the end of 2022, with 0 persons to include in December, analyses were done for August up to and including November.

#### Behavioural change

To describe trends in behaviour among GBMSM and transgender persons, we plotted the numbers and proportions of consultations in which each sexual behaviour variable was reported, and the number of tests and positivity rates for chlamydia, gonorrhoea and infectious syphilis per month. To assess a possible significant decrease in risk behaviour (p < 0.05), we compared the proportions in June, July and August (outbreak months) to May (pre-outbreak month) using chi-square tests. For non-normally distributed continuous variables the Mann–Whitney U test was used. All analyses were done in R version 4.2.0.

## Results

### Epidemiology

Of all persons diagnosed with mpox in the Netherlands, nearly all (99%; 1,275/1,293, for one person sex was unknown) were male at birth, of whom 94% (1,197/1,275) reported to have sex with men. Of those for whom information about gender identity was known, 98% identified as a man (1,101/1,123). The median age was 37 years (interquartile range (IQR): 31–45). Of those eligible for childhood smallpox vaccination (born < 1976 in the Netherlands or < 1980 in another country) and with complete smallpox vaccination information, only 62% (170/276) reported to have received this vaccination in the past. Of those with information about PEP vaccination status for mpox, 4% (52/1,198) reported to have received PEP, but only in two instances it was given at least 14 days before the date of symptom onset. Of those with a known PPV status, 9% (69/795) reported to have received PPV at any time during the outbreak. In 23 instances the last vaccination was given at least 14 days before the date of symptom onset. Of the persons diagnosed with mpox, 51% (660/1,294) originated from the Amsterdam region. Demographical, epidemiological and clinical characteristics of the 294 more recently diagnosed persons were comparable to the first 1,000 cases. For further details on the characteristics of those 1,000 cases, we refer to van Ewijk et al. [9].

### Vaccination

In total, 39,657 invitations for vaccination were sent out by the 25 PHS in the Netherlands. Overall, 29,851 PPV doses were administered between 25 July 2022 and 30 April 2023. Consent to share vaccination information with the RIVM was given for 26,993 vaccinations (90.4%). In total, 17,792 first vaccinations were given, and 11,846 second vaccinations. A total of 213 vaccinations had an unknown ranking number. These first and second PPV doses were administered to 18,684 unique persons, predominantly identifying as men. Background characteristics of the vaccinated persons can be found in Table 1.

**Table 1:** Background characteristics of people vaccinated against mpox, the Netherlands; 25 July 2022–30 April 2023 (n = 18,684)

	n	% of total unique persons
Total	18,684	100
Gender identity		
Man	11,886	63.6
Woman	63	0.3
Other (non-binary, genderfluid, agender etc.)	5,253	28.1
Unknown	1,482	7.9
Age category (year of birth)		
> 1994	2,492	13.3
1985–1994	5,479	29.3
< 1985	10,670	57.1
Unknown	43	0.2
Childhood smallpox vaccination^a^		
Yes	4,369	23.4
No	14,021	75.0
Unknown, born ≥ 1976 and < 1980	129	0.7
Unknown, year of birth unknown	165	0.9
Previous PEP vaccination		
Yes	27	0.1
No	16,400	87.7
Unknown	2,257	12.1
Vaccination indication		
1a. GBMSM and transgender persons using HIV-PrEP via an SHC	5,779	30.9
1b. GBMSM and transgender persons on the waiting list for the HIV-PrEP pilot via an SHC	1,123	6.0
1c. GBMSM and transgender persons using HIV-PrEP via a general practitioner	2,556	13.7
2. GBMSM and transgender persons living with HIV at high-risk of mpox	3,051	16.3
3. Other persons at high risk of mpox via an SHC	3,106	16.6
Unknown^b^	2,542	13.6
Other^c^	527	2.8

GBMSM: gay, bisexual and other men having sex with men; PEP: post-exposure prophylaxis; PrEP: pre-exposure prophylaxis; SHC: sexual health centre.

^a^ For the analyses, we assumed that people born < 1976 with an unknown childhood smallpox vaccination status had received this vaccination as a child (n = 815), while childhood smallpox vaccination status was set to ‘No’ for everybody born ≥ 1980. For those born between 1976 and 1980, and those with missing year of birth, we used smallpox vaccination status as reported.

^b^ No information on indication group, or persons who did not give consent for sharing their vaccination information with the Dutch National Institute for Public Health and the Environment.

^c^ Other indication than predefined indication groups. These are not taken into account in the analyses of the uptake and vaccination coverage since these persons were not specifically invited.

Due to rounding, percentages might add up to 99.9% or 100.1% instead of 100%.

**Figure 1:**
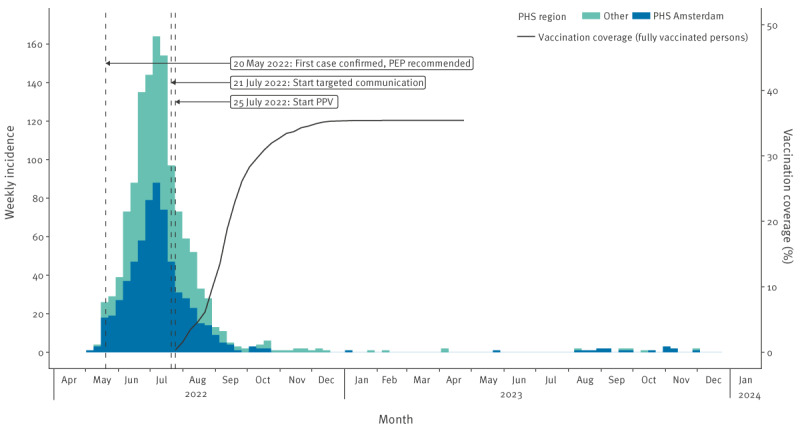
Epidemic curve by date of mpox symptom onset and overall vaccination coverage, the Netherlands; 25 July 2022–30 April 2023 (n = 1,294)

PEP: pre-exposure prophylaxis; PPV: primary preventive vaccination; PHS: public health service.

The overall uptake of vaccination (at least one dose received) was 45.8% (18,157/39,657), with 35.4% (14,048/39,657) being fully vaccinated by 30 April 2023 and considerable variation between the different indication groups (Table 2). The highest uptake and vaccination coverage were found in GBMSM and transgender persons using HIV-PrEP, either via an SHC (indication group 1a) or GP (indication group 1c). The vaccination coverage in relation to the epidemic curve is shown in Figure 1.

**Table 2:** Invited persons, uptake and mpox vaccination coverage per indication group, the Netherlands; 25 July 2022–30 April 2023 (n = 39,631)

Vaccination indication	Invited persons^a^	Partly vaccinated persons (one MVA-BN, no childhood smallpox vaccination)	Fully vaccinated persons		% Uptake^c^	% Vaccination coverage^d^
			Two MVA-BN^b^	One MVA-BN + childhood smallpox vaccination		
1a. GBMSM and transgender persons using HIV-PrEP via an SHC	8,702	954	4,013	812	66.4	55.4
1b. GBMSM and transgender persons on the waiting list for the HIV-PrEP pilot via an SHC	2,776	194	819	110	40.5	33.5
1c. GBMSM and transgender persons using HIV-PrEP via a general practitioner	4,811	515	1,624	417	53.1	42.4
2. GBMSM and transgender persons living with HIV at high risk of mpox	9,858	1,403	1,421	227	30.9	16.7
3. Other persons at high-risk of mpox via an SHC	13,484	658	2,033	415	23.0	18.2

GBMSM: gay, bisexual and other men having sex with men; MVA-BN Modified Vaccinia Ankara-Bavarian Nordic; PrEP: pre-exposure prophylaxis; SHC: sexual health centre.

^a^ For 26 invitations it was unknown which indication group the person belonged to.

^b^ Including persons who had received a childhood smallpox vaccination but who were reported to be immunocompromised and therefore needed two MVA-BN for optimal protection.

^c^ Uptake: proportion of invited persons receiving at least one dose MVA-BN during the current campaign, i.e. (number of party vaccinated + fully vaccinated persons) / number of invited persons.

^d^ Vaccination coverage: proportion of invited persons being fully vaccinated, i.e. number of fully vaccinated persons / number of invited persons.

If the date of symptom onset was unknown, we used the earliest of either sampling date or notification date to the PHS. A bin width of 7 days was used, and data were grouped by PHS Amsterdam vs the rest of the Netherlands. Vaccination coverage data was only available until 30 April 2023.

### Vaccine effectiveness

Between 8 August–4 December, a total of 181 persons diagnosed with mpox were notified to the RIVM. Of those, 19 were excluded as they did not meet inclusion criteria to calculate the VE (i.e. not male, did not report to have sex with men, or vaccination status unknown). Table 3 shows the 162 included cases and VE per week. We estimated overall VE of full vaccination against laboratory-confirmed symptomatic mpox disease to be 68.2% (95% confidence interval: 4.3–89.5). The three fully vaccinated cases all received two PPV doses during the current vaccination programme.

**Table 3:** Mpox cases with known vaccination status, vaccination coverage and effectiveness (as estimated by the screening method), by week, the Netherlands, August–November 2022 (n = 162)

Week number	Start date of week	Cases^a^			Population^b^		% Vaccine effectiveness
		Fully vaccinated cases	Total number of cases	Proportion of cases fully vaccinated	Fully vaccinated persons in the population	Proportion of population fully vaccinated^c^	
32	8 Aug	0	52	0.0	139	0.4	100
33	15 Aug	0	31	0.0	629	1.6	100
34	22 Aug	0	27	0.0	1,364	3.4	100
35	29 Aug	0	13	0.0	1,826	4.6	100
36	5 Sep	0	10	0.0	2,441	6.2	100
37	12 Sep	0	6	0.0	3,921	9.9	100
38	19 Sep	1	4	25.0	5,334	13.5	−114.5
39	26 Sep	0	0	NA	7,467	18.8	NA
40	3 Oct	0	2	0.0	9,048	22.8	100
41	10 Oct	1	5	20.0	10,370	26.1	29.4
42	17 Oct	1	6	16.7	11,213	28.3	49.3
43	24 Oct	0	2	0.0	11,743	29.6	100
44	31 Oct	0	1	0.0	12,249	30.9	100
45	7 Nov	0	0	NA	12,668	31.9	NA
46	14 Nov	0	1	0.0	12,947	32.6	100
47	21 Nov	0	1	0.0	13,251	33.4	100
48	28 Nov	0	1	0.0	13,358	33.7	100
**Overall**		**3**	**162**	**1.9**	NA		**68.2**

NA: not applicable.

^a^ Cases: persons diagnosed with mpox.

^b^ Population: all gay, bisexual and other men having sex with men and transgender persons invited for primary preventive vaccination (n = 39,657).

^c^ A 14-day delay was considered to allow time for vaccine-induced protection (e.g. persons vaccinated in week 30 are shown as fully vaccinated in week 32).

### Behavioural change

**Figure 2:**
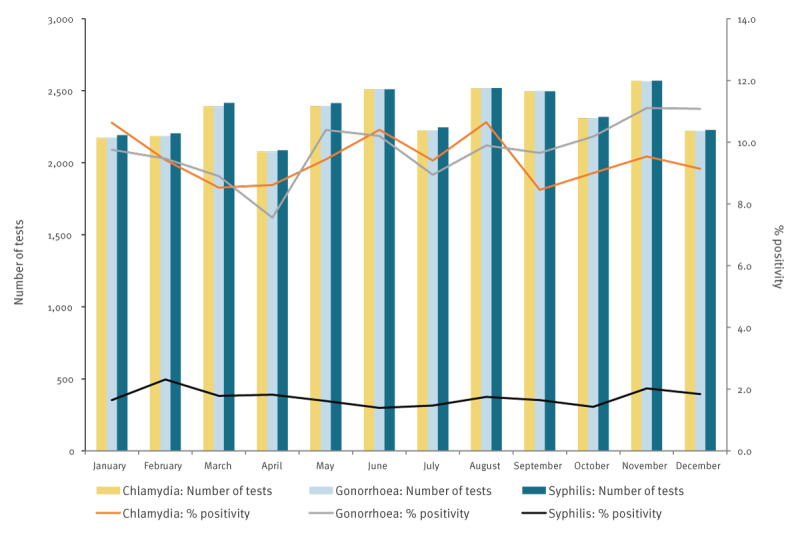
Number of chlamydia, gonorrhoea and syphilis tests and test positivity in GBMSM and transgender persons using HIV-PrEP, the Netherlands, 2022 (n = 28,570 consultations)

GBMSM: gay, bisexual and other men having sex with men; PrEP: pre-exposure prophylaxis.

In 2022, 73,885 consultations were registered among GBMSM and transgender persons at SHC, of which 28,570 (38.7%) were HIV-PrEP pilot consultations. The median age of included persons consulting SHC for HIV-PrEP was 37 years (IQR: 30–49). In this group, no trend was seen in STI rates; see Figure 2 for the number of STI tests and test positivity. Supplementary Tables S6-S8 contain the corresponding results of the chi-square analyses. Chi-square analyses of behavioural variables revealed a significant increase in the proportion of consultations in August vs May where group sex in the past 6 months was reported (43% vs 38%; p < 0.001); these analyses are appended in Supplementary Figure S2 and Supplementary Table S2. Also, a small but statistically significant decrease in the proportion of consultations in June vs May where sex work in the past 6 months was reported (2.4% vs 3.3%; p = 0.049); for the detailed visualisation of the data and results of the chi-square analyses we refer to Supplementary Figure S5 and Supplementary Table S5. No trend was seen in the number of sex partners, chemsex or condomless anal sex. Detailed analyses of these variables can be found in the Supplement.

## Discussion

The PPV programme introduced in the Netherlands in response to the mpox epidemic was targeted at persons at highest risk of mpox infection. It resulted in an overall vaccination coverage of 35.4% by 30 April 2023, with differences between the different indication groups. In addition, we found a VE of full vaccination against laboratory-confirmed symptomatic mpox disease of 68.2%, which is similar to estimates from other studies. For one MVA-BN dose, VE estimates in the literature range from 36 to 86% [18-22], and for two doses from 66% to 89% [20-22]. Persons who received a childhood smallpox vaccination were also included in the referenced studies, however, they would still receive two MVA-BN doses. Those persons would have been considered fully vaccinated after one MVA-BN vaccination in our definition. Therefore, it was to be expected that our estimated VE is a bit lower than the estimate for two doses MVA-BN. Also, it should be noted that the confidence interval of our estimate was wide (4.3%–89.5%) due to the low number of cases, hampering interpretation. However, it is still consistent with estimates that used more data [18-22]; thereby providing a confirmation of these findings.

Generally, a good immune response is seen 2 weeks after the first dose of MVA-BN, with seroconversion seen in 91% of subjects in a study by Pittman et al. [23]. However, antibody titres increased significantly after a second vaccination with MVA-BN administered 4 weeks after the first vaccination [23]. When also taking an incubation period of 8–9 days into account [24,25], it may take several days to weeks before a significant effect of the vaccination programme on infections could be noticed. In addition, the timing and speed with which the vaccination programme is implemented also influences its impact. Although efforts were made to implement a PPV programme as soon as possible, it started in the Netherlands after the peak of the outbreak. By the time PPV would be effective, the incidence was already down to ca five cases per day, making it unlikely that PPV contributed significantly to the decline of the outbreak. Other studies using real world data, for example in Spain and the United Kingdom (UK), also indicate that PPV started at or after the peak of the outbreak [26,27]. The UK study estimated that many more cases could have been averted if vaccination had started earlier [26]. In the US, where vaccination started earlier relative to the peak of the outbreak, the effect of PPV was larger [28].

Unfortunately, the effect of PEP given to high-risk contacts of mpox cases could not be assessed, as national data are lacking. It is unlikely that PEP contributed substantially given that the incubation period of mpox is about equal to, or possibly even shorter than, the time to generate vaccine-induced immunity [23,24]. Furthermore, as it takes time to identify high-risk contacts of an mpox case, invite them for vaccination and administer the vaccine, the impact of PEP to prevent mpox after exposure is likely to be negligible [29]. However, PEP vaccinations may have contributed to the overall effect of mpox vaccination, as they were administered to the persons at highest risk of mpox infection. Therefore, when available, PEP is a logical intervention to implement during an outbreak to bridge the time until a PPV programme is established.

Correct calculation of the uptake, vaccination coverage and assessing VE was complicated due to limitations regarding the quality of data and lack of data on denominators. Identifying and personally inviting the population at risk through multiple healthcare providers was challenging. In addition, some people used an alias, and privacy guidelines prohibited direct comparison of persons possibly invited multiple times. For example, in the second round of a repeat behavioural survey about mpox conducted from 1 July to 3 August 2023 among 2,358 GBMSM in the Netherlands, 16.3% of the participants invited for vaccination reported to have received more than one invitation for vaccination (personal communication, J. de Wit, 28 August 2023). Furthermore, the exact number of invitations sent out by GPs and HIV healthcare providers were not known. Finally, it is known that several HIV healthcare providers invited all their patients instead of only those at highest risk of mpox, which could explain the lower PPV uptake in this group. Overall, the denominator is likely overestimated, but it is unknown to what extent. On the other hand, the number of administered vaccinations might be underestimated, since not all data were available due to privacy constraints. Altogether, we believe we may have underestimated the uptake, vaccination coverage and VE. Thus, our results emphasise the importance of a vaccination register with national coverage and adequate privacy assurance, to facilitate adequate monitoring and evaluation of future vaccination programmes in outbreak settings.

Our observations regarding sexual activity differ from reports from other sources. In the first round of a repeat behavioural survey about mpox conducted from 29 July to 30 August 2022 among 2,460 GBMSM in the Netherlands [30], half (50.4%) of the participants had reduced their number of partners since the start of the outbreak, and two-thirds (65.5%) had avoided sex on premises venues (personal communication, J. de Wit, 28 August 2023). Similar behavioural adaptations to mpox were observed among GBMSM in the US [31]. However, when examining our data on SHC visits, we could not identify comparable changes in (proxies for) behaviour, nor in positivity rates of STI. Most behavioural indicator variables remained relatively stable in 2022. The changes observed simply entailed a minor reduction in reports of sex work in the past 6 months in June compared with May, and a slight increase in reports of group sex in the past 6 months in August vs May. Although these findings align with the hypothesis of decreased sexual activity during the outbreak followed by a re-engagement in sexual activity as the outbreak waned, the extent of these changes in our data was extremely limited.

The fact that, at each point in time, our behavioural indicator variables consisted of reports for the past 6 months (rather than the last month, for instance) could have hindered the detection of more noticeable changes in behaviour, particularly if GBMSM’s behavioural adaptations primarily occurred during the brief period when high numbers of mpox cases were reported. Ad hoc surveys using shorter recall periods, asking about more recent behaviour and changes in behaviour, would be more informative to answer these questions. Also, HIV-PrEP users on whom the analyses were based were those who regularly visit SHC and may not be fully representative of the entire population of GBMSM at risk of mpox. However, this selection was the best possible with regard to the available data.

A likely contributor to the declining course of the outbreak may be infection-acquired immunity in a substantial proportion of those most at risk. This is also in line with results from several modelling studies that conclude that immunity due to infection in the most sexually active GBMSM may have been an important driver of the decline [32-34]. However, these studies also acknowledge that behavioural changes might have had a substantial impact on the course of the epidemic. Modelling results using data on Dutch mpox cases before the start of the PPV programme indicate that increased immunity due to infections may have limited further growth of the mpox epidemic but could not exclusively explain the fast decline after the peak [34]. The model suggests that behavioural adaptations among GBMSM (including reduction in the number of sexual partners) accelerated the decrease observed before the start of PPV. It may also explain the observed vaccine uptake, which was notably lower than what had been expected based on previous studies investigating mpox vaccine acceptance among GBMSM in the Netherlands where 70–85% of GBMSM in the period from July to September 2022 said that they would be willing to accept mpox vaccination [35,36]. The perceived sense of urgency may have been low at the time people were invited for PPV, especially for the second dose, as the outbreak was already declining. This may have led to a lower vaccine uptake and vaccination coverage than expected. Fast implementation of future vaccination programmes is essential to be effective in preventing new cases [37]. Immunity due to infection and vaccination among the most sexually active GBMSM reduces the risk of resurgence of mpox [32,34] and will protect against (severe) disease. The mpox vaccination programme in the Netherlands continued in 2023 and took newly identified risk factors, such as visiting sex venues, into account [30,38].

## Conclusion

We hypothesise that infection-induced immunity in the groups at highest risk is an important factor explaining the fast decline of the mpox outbreak. The role of behavioural change could unfortunately not be adequately studied with the available data, but based on other studies it should not be ignored. It is unlikely that PPV was the main driver of decline of the mpox outbreak, as coverage only started to increase after the decline was well underway. PEP vaccination is likely to have contributed, but lack of nationwide data prevented us from studying this. Regardless of this, timely vaccination may play an important role to prevent future outbreaks, especially since the VE is expected to be relatively high. Continued vaccination programmes are needed to offer vaccination to new people meeting the criteria for vaccination or recently arrived migrant GBMSM. Surveillance of mpox, studies on behaviour and evaluation of the vaccination programmes, including assessment of VE, are important to inform future mpox prevention and control.

## Supplementary Data

226000 Supplement
